# Pros and Cons of Aspirin Prophylaxis for Prevention of Cardiovascular Events in Kidney Transplantation and Review of Evidence

**DOI:** 10.1155/2019/6139253

**Published:** 2019-05-16

**Authors:** Muhammad Abdul Mabood Khalil, Muhammad Shahab Ud Din Khalil, Said Sayed Ahmed Khamis, Sartaj Alam, Rajendra Govindrao Daiwajna, Ahmed Suleman Rajput, Mohammed M. Alhaji, Vui Heng Chong, Jackson Tan

**Affiliations:** ^1^RIPAS Hospital, Bandar Seri Begawan BA1710, Brunei Darussalam; ^2^Department of Cardiology, Lady Reading Hospital, Postal Code, 25000, Peshawar, Pakistan; ^3^Faculty of Medicine, Menoufia University, Egypt; ^4^Department of Nephrology, Lady Reading Hospital, Postal Code 25000, Peshawar, Pakistan; ^5^PAPRSB Institute of Health Sciences, Universiti Brunei Darussalam, Brunei Darussalam; ^6^Department of Medicine, RIPAS Hospital, Bandar Seri Begawan BA1710, Brunei Darussalam; ^7^Department of Renal Medicine, RIPAS Hospital, Bandar Seri Begawan BA1710, Brunei Darussalam

## Abstract

Kidney transplant recipients have traditional and nontraditional risk factors which can lead to coronary artery disease and sudden death with a functional graft loss. Aspirin has been used traditionally for prevention of cardiovascular and cerebrovascular accidents. It has beneficial effects in secondary prevention of cardiovascular events in general population. Its use for primary prophylaxis is still disputed. Bleeding and theoretical risk of nephrotoxicity are the major concerns about its use. The data on aspirin in kidney transplant population is sparse. This review will focus on various pros and cons of aspirin use for prevention of cardiovascular events in kidney transplant recipients and a way forward.

## 1. Introduction

Cardiovascular disease is the leading cause of morbidity and mortality in kidney transplant recipients (KTR). The age of KTR is increasing over time from 35-45 years in 1988 to 50-64 in 2012 [1]. The increasing age is associated with more accumulation of comorbidities such as diabetes, hypertension, dyslipidemia, and atherosclerosis. The incidence of myocardial infarction after kidney transplantation is 4.7-11.1% [2]. Cardiovascular disease is the leading cause of functional graft loss and it accounts for 30% of overall mortality [3]. Aspirin is widely used for prevention of cardiovascular and cerebrovascular events in the general population. In this review, we will discuss the use of aspirin in primary and secondary prophylaxis for cardiovascular events and its pros and cons in KTR.

## 2. Mechanism of Action of Aspirin

Aspirin inhibits platelet function by acetylation of the platelet cyclooxygenase (COX) [3]. Aspirin is an approximately 150- to 200-fold more potent inhibitor (constitutive) isoform of the platelet enzyme (COX-1) than the (inducible) isoform (COX-2) which is expressed by cytokines, inflammatory stimuli, and some growth factors. As a result, the dose for inflammatory conditions is remarkably high as compared to antiplatelet activity which is around 100 mg/day [4].

## 3. Concerns about Aspirin Use

Nonsteroidal anti-inflammatory medications (NSAIDs) are well known for nephrotoxicity, gastritis, and bleeding. Aspirin, being a NSAID, can also potentially cause these complications. Nephrotoxicity in the setting of kidney transplantation is even more important as the recipient only has one functional kidney. We will review these potential complications in this section.

### 3.1. Nephrotoxicity of Aspirin

Previous studies have shown conflicting results about the use of aspirin and the risk of chronic kidney diseases. Some earlier studies have shown that the use of aspirin is associated with chronic kidney disease [5–7]. Some studies implicated acetaminophen and phenacetin in the development of CKD but not aspirin [8, 9]. Other studies in healthy people did not find any association between aspirin and nephrotoxicity. A study in healthy physicians did not find any correlation between aspirin and other nonsteroidal anti-inflammatory medications and the development of chronic kidney disease [10–12]. Similarly a study on healthy nurses failed to show any association between NSAIDs and the development of chronic kidney disease [13]. Various studies were done on aspirin and its effects on proteinuria and glomerular filtration rates. Multiple randomized controlled trials on aspirin in diabetic patients were not associated with decrement in GFR or albuminuria [14–16]. Another randomized controlled trial on diabetic patients showed significant reduction of proteinuria in 24 hours by using aspirin-dipyridamole [17]. In view of these studies, one can assume that aspirin has negligible nephrotoxicity.

Aspirin has been used for prevention of renal vein thrombosis in KTR. In the majority of these studies, no adverse outcome was observed in terms of graft dysfunction. Aspirin has been shown to improve graft survival in a retrospective study and a meta-analysis [18, 19]. In other studies, the use of aspirin did not improve graft survival but at the same time did not have any adverse effect on graft function. Ali H et al. did not find any beneficial effects of aspirin on improving graft survival and found that it has a negligible effect on kidney allograft function as compared to those who were not on aspirin [20]. In a similar study, aspirin reduced the rate of early graft thrombosis but did not improve renal function or graft survival. However, a trend of lower rate of chronic allograft nephropathy was observed in this study [21]. One can assume from all these studies that the risk of nephrotoxicity with aspirin is insignificant. The summary of these studies has been shown in Table 1.

### 3.2. Bleeding

Low-dose aspirin has been associated with increased risk of bleeding in the general population [41, 42]. Gastrointestinal bleeding [43, 44], intracranial bleeding [44], and postoperative surgical site hemorrhage are common in KTR [44]. Cumulative incidence of hospitalization for gastrointestinal bleeding in KTR is 334 events per 100,000 patient years. The incidence of major nontraumatic bleeding has been reported as 3.5% in KTR as compared to 0.4% in normal population [44]. The GI endoscopic procedures were 15-fold higher than in the general population [44]. The data on bleeding due to low-dose aspirin and its relation with bleeding in KTR is limited [19]. Our literature review found few studies on the prevention of renal vein thrombosis and allograft biopsies. These studies reported mixed results for the risk of bleeding with aspirin. Robertson et al. found major bleeding in 2.7% of cases in a retrospective analysis for the use of aspirin in renal vein thrombosis [22]. In another study on prevention of renal vein thrombosis, postbiopsy macroscopic hematuria was 9 percent in the aspirin treated group and 7 percent in the control group [22]. Hachem et al. in a case control study found no difference at postoperative surgical site hemorrhage [23]. In a retrospective analysis, KTR who were on dual antiplatelet because of coronary artery disease have more blood transfusion as compared to those who were not on dual antiplatelet (30.3% vs. 15.7%) [24]. Requirements for transfusions was also reported in another retrospective study in patients who were on dual antiplatelet. However, on multivariate logistical regression analysis it was not significant [25]. Aspirin in combination with anticoagulants can lead to significant bleeding [26]. There are couple of case series on allograft biopsies and risk of bleeding due to aspirin. Atwell et al. studied the incidence of bleeding after 15,181 percutaneous biopsies of various organs (including kidney) and its association with aspirin. They found no difference in major bleeding, if aspirin was taken within 10 days before kidney biopsy [27]. Baffour et al. [28] analyzed 6,700 renal allograft biopsies and compared various durations of aspirin exposure in KTR and their impact on bleeding. They compared no aspirin exposure in 10 days and exposure of aspirin in 8-10, 4-7, and 0-3 days. They found that the risk of bleeding was more with aspirin exposure within 0-3 days. Interestingly Lee et al. in their retrospective analysis of kidney biopsies (including allografts) showed no major bleeding in patients on aspirin [29]. The data on aspirin and risk of gastrointestinal and intracranial bleeding is sparse. Keeping in mind the risk of aspirin related gastrointestinal bleeding in the general population and 15-fold higher chance of gastrointestinal endoscopic procedure in KTR, this risk cannot be ignored. It is important to evaluate the risk of bleeding in KTR, who are being considered for aspirin prophylaxis.  Table 2 shows a summary of all these studies.

## 4. Aspirin Prophylaxis

### 4.1. Primary Prophylaxis in KTR

Various models have been used to predict cardiovascular events in the general population. These include the Framingham Risk Score, the Reynolds Risk Score, the Prospective Cardiovascular Münster Heart Study (PROCAM), the Systematic Coronary Risk Evaluation system (SCORE), and the QRISK 1 and 2 [45–50]. Recently, U.S. Preventive Task Force has published guidelines for predicting cardiovascular risk using pool cohort equation and primary prophylaxis with aspirin [51]. Risk factors for cardiovascular events in KTR are different from those in the general population. Nontraditional factors play an important role in causing cardiovascular events in this population. These factors include albuminuria, anemia, and graft rejection [52], time on dialysis before transplantation [53], immunosuppressive medications [54], and elevated homocystine [55]. These factors are usually not taken into account in various risk scores. The Framingham Risk Model underestimated cardiovascular events in KTR [56, 57]. The American Heart Association (ACC/AHA) pooled cohort equations to predict 10-year risk have not been validated in KTR to predict cardiovascular risk in this specific group of patients. Recently Heleniak Z et al. retrospectively analyzed various scores for cardiovascular risk prediction in KTR and found that the QRISK2 and Pol-SCORE scales seem to be the most predictive in assessing CV risk in KTR as compared to PROCAM and Framingham [58]. Soveri et al. used a 7-year risk model for KTR [59]. They predicted major cardiovascular events using a seven-variable model including age, previous coronary heart disease, diabetes, low-density lipoprotein, creatinine, number of transplants, and smoking [59]. A systematic review analyzed metrics of model performance and evaluation of bias in KTR and found room for improvement for accurate prediction of cardiovascular risk [60]

Evidence for aspirin use in KTR for primary prophylaxis is still lacking. Guidelines differ in the recommendation of aspirin use in primary prophylaxis. The United States Preventive Services Task Force recommends low-dose aspirin for the primary prevention of cardiovascular disease in adults aged 50-59 years who have a 10% or greater 10-year CVD risk [51]. Candidates for aspirin should not be at increased risk of bleeding, life expectancy should be greater than at least 10 years, and they should be willing to take it regularly. For those aged 60-69, the decision is on an individual basis. If the 10-year CVD risk is greater than 10% and there is no risk of bleeding, then low-dose aspirin should be considered. The evidence for low-dose aspirin prophylaxis in patients younger than 50 years old or older than 70 years is not enough, and the pros and cons of its use are not known. Task Force recommended American Heart Association recommendation pooled cohort equations to predict hard atherosclerotic cardiovascular events (defined as nonfatal myocardial infarction, coronary heart disease death, and fatal or nonfatal stroke) [61]. Calculators assess the risk using various variables including age, gender, race, total cholesterol, HDL-cholesterol, systolic blood pressure, diastolic blood pressure, treatment for high blood pressure, diabetes, and smoking [61]. Unfortunately, there is no validation of ACC/AHA pooled cohort equation in KTR. The American Diabetic Association recommends that 75–162 mg/day may be considered as a primary prevention strategy in those with type 1 or type 2 diabetes who are at increased cardiovascular risk. This includes most men and women with diabetes aged ≥50 years who have at least one additional major risk factor (family history of premature atherosclerotic cardiovascular disease, hypertension, dyslipidemia, smoking, or albuminuria) and are not at increased risk of bleeding [62]. In contrast, the Canadian Cardiovascular Society guideline does not recommend primary prophylaxis with aspirin [63]. Similarly in the UK, aspirin is not recommended for primary prevention of cardiovascular events [64]. No randomized trials to date have evaluated the primary use of aspirin for primary prophylaxis in dialysis or transplant patients. As a result firm recommendations cannot be made. Because of lack of evidence, the use of aspirin in the setting of renal dysfunction has been minimal. Review of National Cardiovascular Data ACTION (Acute Coronary Treatment and Intervention Outcomes Network) in ST elevated myocardial infarction and non-ST elevated myocardial infarction showed lesser use of aspirin with worsening CKD [65]. Similarly, Berger and his colleagues found that end stage renal disease (ESRD) patients with myocardial infarction were less likely to receive aspirin, beta blocker, or angiotensin converting enzyme inhibitor as compared to patients without ESRD. The benefit of these therapies on 30-day mortality was similar among ESRD patients and non-ESRD patients [66]. A recent trial on hypertension (Hypertension Optimal Treatment or HOT) [67] randomly assigned patients with diastolic hypertension to aspirin 75 mg or placebo. Statistical analysis detected a 66% reduction (95% CI, 33 to 83) in major adverse cardiovascular events and a 49% reduction (95% CI, 6 to 73) in mortality, respectively, among the subgroup with baseline eGFR 45 mL/min. Use of aspirin in chronic kidney disease patients reduced in patients' mortality to 64.3-80% across all quartiles of creatinine clearance[68]. A retrospective analysis of acute coronary syndrome patients showed that use of aspirin was associated with a decreased rate of ST-segment elevation myocardial infarction in patients with GFR 60 mls/min [69]. There are few retrospective studies and a meta-analysis in KTR, where aspirin prophylaxis was used for the prevention of renal vein thrombosis [18–20]. However, not all of these studies looked at the cardiovascular mortality. A meta-analysis found that aspirin reduces major adverse cardiovascular events or mortality (2 studies; RR: 0.72, 95% CI: 0.59 to 0.88) in KTR [19]. However, there was no randomized control trial looking for major adverse cardiovascular events in this meta-analysis. Post hoc analysis of the FAVORIT (Folic Acid for Vascular Reduction in Transplantation) study on aspirin failed to show reduction in cardiovascular events [70]. Kidney Disease Improving Global Outcomes (KDIGO) 2009, practice guidelines for prevention of cardiovascular events in kidney transplant recipients with diabetes or cardiovascular disease, suggest use of low-dose aspirin based on very poor quality of evidence [71]. Recently two randomized control trials were published. ASCEND (a Study of Cardiovascular Events in Diabetes) was a randomized trial to assess the efficacy and safety of enteric-coated aspirin at a dose of 100 mg daily vs. placebo, in diabetics without any cardiovascular disease at trial entry [42]. The trial showed significant reduction in cardiovascular events but with more incidence of major bleeding. Thus the beneficial effect of aspirin was negated by major bleeding. Similarly in the ASPREE (Aspirin in Reducing Events in the Elderly) trial, the use of low-dose aspirin as a primary prevention in older adults resulted in a significantly higher risk of major hemorrhage without any significant reduction in cardiovascular diseases as compared to placebo [72]. Keeping these facts, along with the lack of a randomized control trial in KTR, in mind, firm recommendation cannot be made for use of aspirin in primary prevention.

### 4.2. Secondary Prophylaxis in KTR

Patients who suffered from acute coronary syndrome or ischemic strokes are always at risk of a second cardiovascular event. Percutaneous intervention stabilizes acute events only. Aspirin therapy is needed for further prevention of events. The role of aspirin in reducing CVD mortality and repeat events after acute myocardial infarction was first demonstrated in the second International Study of Infarct Survival (ISIS-2) trial [73]. After ISIS-2 many trials confirmed the beneficial effects of aspirin. The Antithrombotic Trialists' Collaboration analyzed 16 trials of long-term aspirin use and found it beneficial for secondary prevention [74]. Cardiovascular disease is the leading cause of functional graft loss and it accounts for 30% of overall mortality [3]. Charytan et al. defined coronary artery disease as > 50% stenotic lesion in hemodialysis patients [75]. Other studies defined CAD as ≥ 70% stenotic lesion in pretransplant evaluation of their cohorts [76, 77]. The benefit of aspirin therapy in the setting of acute coronary syndrome and myocardial revascularization procedure has been shown across all spectra of renal dysfunction [78]. The results of studies comparing medical management vs. revascularization showed mixed findings. Studies have shown benefits of revascularization against medical management in chronic kidney disease patients only if there is ≥ 75% stenosis, triple vessel disease, or left main stem disease [79, 80]. All patients with stable coronary artery disease without obstructive lesions or those with obstructive lesions needing intervention should be on aspirin for secondary prophylaxis. KTR who have revascularization with a stent will need dual antiplatelets therapy including aspirin. Various durations for dual antiplatelets studies have been reviewed. Most studies compared either shorter (3-6 months) [31, 32, 81, 82] or longer (18-48 months) [83–85] duration of exposure. Longer duration of dual antiplatelets is associated with less stent thrombosis but with slightly more bleeding risk [84, 85]. In 2012, the American Heart Association (AHA) and American College of Cardiology (ACC) foundation published their recommendations for kidney and liver transplant recipients, which were endorsed by the American Transplant Society [30]. These guidelines recommend dual antiplatelets for 4-12 weeks for bare metal stent and ≥ 12 months for drug eluting stents.

Newer-generation (everolimus/zotarolimus) drug eluting stents are associated with lower risk of thrombosis and coronary events than the older first-generation stents [31, 32, 86]. Keeping these facts in mind, the American College of Cardiology and American Heart Association 2016 guidelines improved the 2012 guidelines which were previously endorsed by American Society of Transplantation. The new 2016 guidelines recommend 6 months of dual antiplatelets therapy in patients treated with drug eluting stent and having stable ischemic heart disease [33]. Subsequent duration of dual antiplatelet therapy after PCI in stable coronary heart disease depends on the risk of bleeding. ACC/AHA2016 guidelines recommend continuation of dual antiplatelet beyond 1 month with bare metal stent and more than 6 months in drug eluting stent in patients who are at low risk of bleeding. Low risk patients include patients having no prior bleeding on dual antiplatelet, having no coagulopathy, and not being on oral anticoagulant [33]. In contrast, in those with high risk of bleeding (on oral anticoagulant, undergoing intracranial surgery, or developing overt bleeding), discontinuation of P2Y12 inhibitor therapy after 3 months may be reasonable [33]. The guidelines for PCI followed by stenting in the setting of acute coronary syndrome (ACS) are slightly different. In this scenario, prolonged dual antiplatelet therapy for 12 months has been found beneficial [34, 35]. Therefore, ACC/AHA2016 guidelines recommend that, for patients with acute coronary syndrome (NSTEMI/STEMI) treated with dual antiplatelets after bare metal stents or drug eluting stent insertion, thienopyridines should be given for at least 12 months [33]. The guidelines further recommend that for patients with acute coronary syndrome who has tolerated dual antiplatelets without a bleeding complication and who are not at high risk of bleeding, continuation of dual antiplatelets beyond 12 month may be reasonable. On the other extreme, for high risk ACS patients (treatment with oral anticoagulation, high risk of bleeding due to intracranial surgery, or development of overt bleeding), discontinuation of P2Y12 inhibitor after 6 months may be reasonable [33]. Patients with ACS who never underwent revascularization or fibrinolytic therapy should be treated with dual antiplatelets for at least 12 months [33, 34, 36]. For those who have tolerated dual antiplatelet therapy and are at low risk of bleeding, continuation of these beyond 12 months is beneficial [33]. For patients with ST elevated myocardial infarction, therapy should be continued for a minimum period of 14 days [33, 36] and ideally at least 12 months [33]. For those who tolerated dual antiplatelet therapy and are at low risk of bleeding complications, ACC/AHA recommends antiplatelets continuation beyond 12 months.

The timing of transplant surgery and other noncardiac surgeries in patients on antiplatelets therapy needs to assess risk vs. benefit of stopping antiplatelet and doing that surgery. It is wise to have a multidisciplinary meeting including cardiologist, anesthetist, surgeon, and transplant physician before taking a decision. For patients who need percutaneous intervention (PCI) and are planning for transplantation within 1 year, the 2012 guidelines [30] recommend angioplasty with bare metal stenting followed by 4-12 weeks of dual antiplatelets. For patients who have drug eluting stents needing an urgent surgery and at high risk of bleeding, guidelines recommend holding thienopyridine for 5 days and continuing aspirin preoperatively [30, 33]. Thienopyridine may be started as early as possible after the surgery [30, 33]. The guidelines also recommend that transplantation surgery within 3 months of bare metal stent and within 12 months of drug eluting stent should not be performed [30]. Because of the lower risk of thrombosis with newer-generation stent [31, 32, 86], 2016 ACC/AHA guidelines recommend waiting for 6 months rather than 12 months in case of drug eluting stent [33]. For all elective noncardiac surgeries, it is wise to wait for 3 months in patients with bare metal stents and 6 months in patients with newer-generation drug eluting stents [33, 37–39].

The recommended daily dose for aspirin is 81 mg (range, 75 to 100 mg) for prevention of secondary prophylaxis [33, 40]. Proton pump inhibitors (PPIs) are recommended in patients with dual antiplatelets with increased risk of bleeding. This includes advanced age and concomitant use of warfarin or nonsteroidal anti-inflammatory drugs (class IIa evidence) [33]. Routine use of PPIs for patients at low risk of bleeding is not recommended (class III, no benefits). Since transplant patients are concurrently using steroids, it is wise to use PPIs for prevention of gastrointestinal bleeding.  Table 3 is showing various recommendations for use of aspirin in secondary prophylaxis.

## 5. Way Forward

While starting aspirin, one has to keep in mind risks versus benefits. The benefits of aspirin can be offset by the associated risk of bleeding. This is of particular concern in KTR, who are being considered for primary prevention with aspirin. KDIGO 2009 practice guidelines for prevention of cardiovascular events in KTR recommend aspirin in patients with diabetes or cardiovascular disease. However, this is based on very poor quality of evidence [71]. The recent trial by the ASCEND Study Collaborative Group found that aspirin use prevented serious vascular events in diabetics. However, the absolute benefits were largely counterbalanced by the bleeding hazard [42]. Similarly the elderly population who received aspirin for primary prevention in recent APREE trial has more bleeding without any benefit [72]. However, both of these studies were done in the general population. KTR are different in terms of cardiovascular risk. Other than traditional risk factors, KTR have many others. Furthermore, cardiovascular events are among the leading causes of functional graft loss. Therefore, there is an urgent need for a randomized control trial on aspirin for its use in primary prevention. Until the evidence is available, it cannot be recommended for primary prevention of cardiovascular events at the moment. On the other hand, aspirin should be used routinely for secondary prophylaxis in KTR. Aspirin has not been shown to be associated with nephrotoxicity in many studies [8–21]. However, like in the general population, bleeding is a genuine concern in KTR. ACC/AHA recommends the assessment of the risk of bleeding using HAS-BLED score (Hypertension, Abnormal renal/liver function, Stroke, Bleeding history or predisposition, Labile INR, Elderly, Drugs/alcohol) [33]. Most of these risk factors are present in chronic kidney disease patients and KTR. A new score for dual antiplatelets called DAPT (Dual Antiplatelet Therapy) Score has also been developed [87]. This score uses various factors including various age ranges, current smoking status, diabetes mellitus, myocardial infarction at presentation, prior PCI or prior myocardial infarction, stent diameter < 3 mm, paclitaxel eluting stent, congestive heart failure or left ventricular ejection fraction < 30%, and saphenous vein graft percutaneous intervention. This score helps with the decision whether to give antiplatelet for a long duration or not. Dual antiplatelet may be suitable in those with DAPT Score greater than 2, as use of dual antiplatelet is associated with less risk of ischemic events and less bleeding risk. On the other hand, with a low DAPT Score of <2, prolonged use of dual antiplatelets causes increased risk of bleeding without reduction in ischemic events.

## 6. Conclusion

Aspirin should be used in established coronary artery disease for secondary prevention. Low-dose aspirin has not been shown to cause nephrotoxicity. The beneficial effects of aspirin are offset by high risk of major bleeding in primary prevention in the general population. Due to lack of evidence at the moment, it cannot be recommended for primary prevention of cardiovascular events in KTR. The risk of bleeding should be assessed in all recipients before starting aspirin. KTR have many risk factors other than the traditional risk factors. There is a need for development of a cardiovascular risk prediction score targeting the kidney transplant population. A randomized control trial is also needed to assess the beneficial effect of primary prophylaxis with aspirin in the kidney transplant population. The final decision on using aspirin should be made after balancing the specific characteristics of each patient taken into account the patient's risk for bleeding and the concomitant pathologies in each case.

## Figures and Tables

**Table 1:** Studies on aspirin nephrotoxicity.

Author	Journal/Year	Study Design / Methods	Finding
Morlans M et al. [5]	Br J Clin Pharmacol / 1990	Case control study/ They studied non-narcotic analgesics taken at least every other day for 30 days or longer and compared with control.	Overall odds ratiobefore the first symptom of renal disease was 2.89 (95% CI, 1.78 to 4.68). The risk increased in relation to the use duration.

Fored CM et al. [6]	N Engl J Med / 2001	Case control study / 926 newly diagnosed chronic kidney disease patients were interviewed and logistic-regression models were used to estimate the relative risks of disease-specific types of chronic renal failure associated with the use of various analgesics (aspirin, acetaminophen)	Use of either of the drug was associated with a 2.5 times increase risk of chronic kidney disease.

Ibáñez L et al. [7]	Kidney Int / 2005	Case control studies / Five hundred and eighty-three cases and 1190 controls were included in the analysis.	Long-term use of any analgesic was associated with an overall odds ratio of 1.22 (95% CI, 0.89-1.66). Risks for aspirin was 1.56 (1.05-2.30). The risk of chronic kidney diasesae stage V associated with aspirin was related to the cumulated dose and duration of use, and it was particularly high among the subset of patients with vascular nephropathy as underlying disease [2.35 (1.17-4.72)].

Perneger TV et al. [8]	N Engl J Med /1994	Case control of study of 716 Chronic Kidney Disaese –V. Control were of similar from Maryland, Virginia, West Virginia, and Washington, D.C.	Authors found increased risk of chronic kidney disease-V in a dose-dependent fashion with acetaminophin. A cumulative dose of 5000 or more pills containing NSAIDs was also associated with an increased odds of ESRD (odds ratio, 8.8). Aspirin was not associated with increased risk of chronic kidney disease.

Sandler DP et al. [9]	N Engl J Med / 1989	Multicenter case-control study to examine the use of analgesic as cause of chronic kidney disease. A total of 554 adults with newly diagnosed kidney disease and 516 matched control subjects selected randomly from the same area of North Carolina.	The risk of renal disease was highest in daily users of phenacetin (odds ratio, 5.11; confidence interval, 1.76 to 14.9, after adjustment for the effects of other analgesics). The risk of renal disease was also increased in daily users of acetaminophen; after adjustment for the use of aspirin and phenacetin, the odds ratio was 3.21 (confidence interval, 1.05 to 9.80). There was no increased risk in daily aspirin users (adjusted odds ratio, 1.32; confidence interval, 0.69 to 2.51).

Kurth at al. [10]	Am J Kidney Dis / 2003	Prospective cohort study of healthy male physicians. Self-reported use of aspirin, acetaminophen, and other nonsteroidal anti-inflammatory drugs (NSAIDs) was classified as never (<12 pills during the study period), 12 to 1,499 pills, 1,500 to 2,499 pills, and 2,500 or greater pills during the study period.	Authors concluded that occasional to moderate analgesic intake of aspirin, acetaminophen, or NSAIDs does not appear to increase the risk for decline in kidney function during a period of 14 years in healthy physicians.

Rexrode KM et al. [11]	JAMA / 2001	Prospective cohort study / Data was taken from the Physicians' Health Study, which lasted 14 years from September 1982 to December 1995 with annual follow-up.	Multivariable analyses adjusted for age; body mass index; history of hypertension, elevated cholesterol, and diabetes; occurrence of cardiovascular disease; physical activity; and use of other analgesics, the relative risks of elevated creatinine level associated with intake of 2500 or more pills were 0.83 (95% confidence interval [CI], 0.50-1.39; P for trend =.05) for acetaminophen, 0.98 (95% CI, 0.53-1.81; P for trend =.96) for aspirin, and 1.07 (95% CI, 0.71-1.64; P for trend =.86) for other nonsteroidal anti-inflammatory drugs. No association was observed between analgesic use and reduced creatinine clearance.

Agodoa LY et al. [12]	Am J Kidney Dis / 2008	Cross-sectional analysis of National Health and Nutrition Examination Survey conducted in 1999-2002. Age-standardized prevalence in habitual analgesic users and non-habitual analgesic users and multivariable-adjusted odds ratios (ORs) were measured.	Habitual analgesic use of single or multiple products was not associated with increased prevalence of albuminuria or reduced eGFR as compared to non-habitual analgesic user.

Curhan GC et al. [13]	Arch Intern Med / 2004	Prospective Health Nurse Study. Informations were gatherered via a mailed questionnaire in 1999 about lifetime use of acetaminophen, aspirin, and NSAIDs and provided blood samples in 1989 and 2000.	Acetaminophen use was associated with an increased risk of a GFR decline of at least 30 mL/min per 1.73 m(2) (P trend =.01) and a GFR decline of 30% or greater (P trend<.001), but aspirin and NSAID use were not.

Okada S et al. [14]	PLoS One. 2016 Jan	Randomized controlled trial (RCT), the Japanese Primary Prevention of Atherosclerosis with Aspirin for Diabetes (JPAD) trial, to evaluate low-dose aspirin as primary prevention for CVD in patients with type 2 diabetes. Patients with negative urine dipstick albumin of the JPAD trial in were followed in a cohort study after the RCT period was completed.	Low-dose aspirin did not increase the risk of positive urine dipstick albumin. There were no significant differences in annual changes in eGFR between the groups (aspirin, -0.8±2.9; no aspirin, -0.9±2.5 ml/min/1.73 m(2)/year).

Hansen HP et al. [15]	Diabetes Care / 2000	Randomized double-blind crossover trial of 17 type 1 diabetic patients with microalbuminuria to study the effect of aspirin on proteinuria	Use of 150 mg ASA daily does not have any impact on albumin excretion rate or glomerulofilteration rate in type 1 diabetic patients with microalbuminuria.

Gaede P et al. [16]	Nephrol Dial Transplant/2003	Randomized, double-blind, crossover trial, of 31 type 2 diabetic patients with elevated levels of AER (>30 mg/24 h) were, in random order, given ASA (150 mg/day) for 4 weeks followed by placebo for 4 weeks with a 2 week washout period or vice versa.	Low-dose treatment with 150 mg aspirin daily does not have any impact on albumin excretion rate or GFR in type 2 diabetic patients with micro- or macroalbuminuria.

Hopper AH et al. [17]	Nephrol Dial Transplant / 1989	Double-blind crossover pilot study to study the effect of administration of aspirin-dipyridamole and reduction of proteinuria in diabetic nephropathy.	24 hour urinary protein excretion significantly reduced during aspirin-dipyridamole administration from a geometric mean (range) of 1.9 (0.4-7.7) g/24 h to 1.4 (0.5-9.9) g/24 h (P vaslue less than 0.05).

Grotz W et al. [18]	Transplantation / 2004	Retrospective, multivariate analysis toassess the effect of low-dose aspirin treatment (100 mg/day) on allograft function and survival of 830 renal transplant recipients.	Allograft survival was significantly longer in patients receiving low-dose aspirin therapy compared with patients receiving no aspirin treatment (n=205, 13.8 +/- 2.6 vs. 7.8 +/- 0.3 years, n=625; adjusted relative risk=0.443, 95% confidence interval [0.323-0.608], P<0.0001).

Cheungpasitporn W et al. [19]	J Nephropathol / 2017	Metanalysis of 19759 KTR	Aspirin reduced the risk of allograft failure (4 studies; RR: 0.57, 95% CI: 0.33 to 0.99), allograft thrombosis (2 studies; RR: 0.11, 95% CI: 0.02 to 0.53), and major adverse cardiac events (MACEs) or mortality (2 studies; RR: 0.72, 95% CI: 0.59 to 0.88), but not allograft rejection (3 studies; RR: 0.86, 95% CI: 0.45 to 1.65) or delayed graft function (DGF) (2 studies; RR: 1.00, 95% CI: 0.58 to 1.72) In KTR. Data on risk of major or minor bleeding were limited.

Ali H et al. [20]	Experimental and Clinical Transplantation / 2017	Retrospective analysis of 82 patients on low-dose aspirin 75 mg once daily who underwent renal transplant between 1 January 2000 and 31 December 2010 from a single center with 65 patients not taking aspirin.	Aspirin use was not significantly associated with creatinine levels (P = .59) after adjusting for other relevant variables.

Murphy GJ et al. [21]	Br J Surg /2001	A prospective consecutive series of 105 cadaveric renal transplants treated with aspirin 150 mg daily for the first 3 months after transplantation was compared with an untreated historical control group (n = 121). Needle protocol core biopsies were performed.	Rate of significant primary allograft thrombosis in patients treated with aspirin (none of 105) compared with that in the control group (six (5 per cent) of 121; P = 0.03) was found. No differences in renal function or 2-year allograft survival between the two groups was found. Aspirin-treated patients had a lower incidence of chronic allograft nephropathy at 1 year than controls, however P value was not significant.

**Table 2:** Summary of studies on aspirin and bleeding risk

Author	Journal / Year	Study Design / Method	Finding
Robertson AJ et al. [22]	Nephrol Dial Transplant / 2000	Retrospective study of effect of 75 mg once a day starting immediately before and continuing for 1 month post-transplant for prophylaxis against renal vein thrombosis.	Out of 480 patients, post biopsy bleeding was present in 8 (1.6%), early post- transplant bleeding in 3 (1%) and re-exploration for bleeding in 3 (1%).

Murphy GJ et al. [22]	Br J Surg / 2001	A consecutive series of 105 cadaveric renal transplants treated with aspirin 150 mg daily for the first 3 months after transplantation was compared with an untreated historical control group (n = 121) for prevention of renal vein thrombosis.	Post biopsy macroscopic hematuria was 9 percent in aspirin treated group and 7 percent in the control group.

Hachem LD et al. [23]	TransplInt / 2017	Case–control study of patients receiving a kidney transplant / To study postoperative surgical-site hemorrhage after kidney Transplantation: incidence, risk factors, and outcomes. Authors also analyzed 86/354 patients who were taking antiplatelet.	Bleeding was not significant in KTR with antiplatelet as compared to those who were not on antiplatelet. [1.26 (0.70, 2.27) P value=0.43]

Weng F et al. [24]	Am J Transplant / 2016	Retrospective cohort study of kidney transplant recipients who received a transplant from 2008-2014 and had a pre-transplant diagnosis of coronary artery disease and receiving dual antiplatelet.	Transfused during transplant hospitalization 30.3% in those on dual antiplatelet as compared to 15.7% who were not on dual antiplatelet. (P value 0.03)

Bailey PD et al. [25]	Austin J Nephrol Hypertens / 2015	A retrospective cohort study of consecutive adult living- and deceased-donor kidney-only recipients. Consecutive adult kidney-only recipients from taking aspirin alone (ASA), ASA and Plavix® (DUA), or no Antiplatelet therapy at the time of transplantation were assessed. The primary outcome was at least one blood transfusion during or within 5 days of transplantation. Secondary outcomes included many including reoperation for bleeding.	Blood transfusion was required in 34.6% within 5 days of kidney transplantation. Perioperative blood transfusion was given in 27.8% of patients in the NONE group, 52.2% of cases in the DUAL group, and42.2% of cases in the ASA group (p<0.01) suggesting an association of ASA and DUAL with blood transfusion on univariate analysis. Antiplatelet therapy, either as DUAL or ASA alone, was not associated with reoperation for bleeding (1.0%, 0.0%, 1.5% p = 0.79)

Shullo MA et al. [26]	Pharmacotherapy / 2002	Retrospective chart review of thirteen patients who had received enoxaparin within 10 days of kidney or kidney-pancreas transplantation. Major bleeding events were defined as intracranial or retroperitoneal bleeding, or a decrease in hemoglobin of greater than 2 g/dl.	Nine (69%) of the 13 patients had confirmed major bleeding events and required blood transfusions.

Atwell TD et al. [27]	AJR Am J Roentgenol. / 2010	The objective of our study was to report the incidence of bleeding after imaging- guided percutaneous core biopsy at a single center using a standardized technique.	The incidence of bleeding in kidney biopsy, 0.7%; highest than the rest of organs. No statistically significant difference in the major bleeding complication rates was seen between patients who took aspirin within 10 days before biopsy compared with those who did not took aspirin.

Baffour FI et al. [28]	Journal of vascular and interventional radiology JVIR / 2017	Retrospective analysis to determine if patient aspirin exposure and timing affect bleeding risk after renal allograft biopsy. Four groups were analyzed which included no aspirin exposure 10 days, exposure within 8-10 days, exposure within 4-7 days and 0-3 days.	Aspirin use was not significantly associated with increased risk of bleeding complication except for use of 325 mg of aspirin within 3 days of biopsy (any complication OR 3.87 [1.12, 13.4], P = .032; major complication OR 6.30 [1.27, 31.3], P = .024).

Lees JS et al. [29]	Clin Kidney J. 2017	Retrospective data review /This study aimed to describe the incidence of major bleeding after biopsy in a single center over a 15-year period and examine factors associated with major bleeding. Aspirin was routinely continued	Aspirin was taken by 327 / 1509 patients. There was no significant increase in the risk of major bleeding (P=0.93).

**Table 3:** Guidelines for use of aspirin for secondary prophylaxis.

Clinical Scenarios	Recommendations
Coronary artery disease stented with first generation bare metal stent	Dual antiplatelets for 4-12 weeks [30]

Coronary artery disease stented with first generation drug eluting stent	Dual antiplatelet for ≥ 12 months [30]

Transplant surgery	Transplant surgery within 3 months of bare metal stent and within 12 months of drug eluting stent should not be performed [30]

Stable coronary artery disease stented with newer-genration (everolimus / zotarolimus) drug eluting stents	Dual antiplatelet therapy for 6 months [31–33]

Coronary artery disease stented with low risk of bleeding and having newer-genration (everolimus / zotarolimus) drug eluting stents or bare metal stent	Guidelines recommend continuation of dual antiplatelet beyond 1 month in baremetal stent and more than 6 months in drug eluting stent in patients who are at low risk of bleeding [33].

Coronary artery disease stented with high risk of bleeding and having newer-genration (everolimus / zotarolimus) drug eluting stents or bare metal stent	Discontinuation of P2Y12 inhibitor therapy after 3 months may be reasonable in those with high risk of bleeding

Patient with acute coronary syndrome (NSTEMI / STEMI) treated baremetal stent or newer generation drug eluting stent	Dual antiplatelet should be given for atleast 12 months [33–35]

Patients with acute coronary syndrome treated with stenting, who has tollerated dual antiplatelets without a bleeding complication, and who are not at high risk of bleeding	Continuation of dual antiplatelets beyond 12 month may be reasoable [33]

Patients with acute coronary syndrome treated with stenting and at high risk of bleeding	Discontinuation of P2Y12 inhibitor after 6 months may be reasonable [33]

Patients with ACS who never underwent revascularization or fibrinolytic therapy	They should be treated with dual antiplatelets for at least 12 months [33, 34, 36].

ST elevated myocardial infarction	Should be continued on dual antiplatlet for a minimum period of 14 days [33, 36] and ideally at least 12 months [33].

Patients planning for transplantation in one year and needing PCI	Angioplasty with bare metal stenting followed by 4-12 weeks of dual antiplatelets [30].

KTR on dual antiplatelets needing emergency surgery	Hold thienopyridine for 5 days and continuing aspirin preoperatively [30, 33].Thienopyridine, may be started as early as possible after the surgery [30, 33].

Waiting time for kidney transplantation and other elective surgery after PCI	Wait for 3 months in case of bare metal stenting and 6 months for drug eluting stenting [33, 37–39].

Dose of aspirin	The recommended dialy dose for aspirin is 81 mg (range, 75 to 100 mg) for prevention of secondary prophylaxis [33, 40]

Proton pumpinhibitors	Proton pump inhibitors (PPIs) are recommended in patients with dual antiplatelets with increased risk of bleeding. This includes advance age, concomitant use of warfarin or non steroidal antiinflammatory drugs (class 2a evidence). Routine use of PPIs in patients at low risk of bleeding is not recommended (class III, no benefits) [33].
